# Pre-hospital Prognostic Factors of Out-of-Hospital Cardiac Arrest: The Difference Between Pediatric and Adult

**DOI:** 10.3389/fped.2021.723327

**Published:** 2021-10-21

**Authors:** Fu-Jen Cheng, Wei-Ting Wu, Shih-Chiang Hung, Yu-Ni Ho, Ming-Ta Tsai, I-Min Chiu, Kuan-Han Wu

**Affiliations:** Department of Emergency Medicine, Kaohsiung Chang Gung Memorial Hospital, Chang Gung University College of Medicine, Kaohsiung City, Taiwan

**Keywords:** out-of-hospital cardiac arrest, pediatric, pre-hospital, advanced airway management, public

## Abstract

The prognosis of out-of-hospital cardiac arrest (OHCA) is very poor. Although several pre-hospital factors are associated with survival, the different association of pre-hospital factors with OHCA outcomes in pediatric and adult groups remain unclear. To assess the association of pre-hospital factors with OHCA outcomes among pediatric and adult groups, a retrospective observational study was conducted using the emergency medical service (EMS) database in Kaohsiung from January 2015 to December 2019. Pre-hospital factors, underlying diseases, and OHCA outcomes were collected for the pediatric (Age ≤ 20) and adult groups. Kaplan-Meier type plots and multivariable logistic regression were used to analyze the association between pre-hospital factors and outcomes. In total, 7,461 OHCAs were analyzed. After adjusting for EMS response time, bystander CPR, attended by EMT-P, witness, and pre-hospital defibrillation, we found that age [odds ratio (OR) = 0.877, 95% confidence interval (CI): 0.764–0.990, *p* = 0.033], public location (OR = 7.681, 95% CI: 1.975–33.428, *p* = 0.003), and advanced airway management (AAM) (OR = 8.952; 95% CI, 1.414–66.081; *p* = 0.02) were significantly associated with survival till hospital discharge in pediatric OHCAs. The results of Kaplan-Meier type plots with log-rank test showed a significant difference between the pediatric and adult groups in survival for 2 h (*p* < 0.001), 24 h (*p* < 0.001), hospital discharge (*p* < 0.001), and favorable neurologic outcome (*p* < 0.001). AAM was associated with improved survival for 2 h (*p* = 0.015), 24 h (*p* = 0.023), and neurologic outcome (*p* = 0.018) only in the pediatric group. There were variations in prognostic factors between pediatric and adult patients with OHCA. The prognosis of the pediatric group was better than that of the adult group. Furthermore, AAM was independently associated with outcomes in pediatric patients, but not in adult patients. Age and public location of OHCA were independently associated with survival till hospital discharge in both pediatric and adult patients.

## Introduction

The prognosis of out-of-hospital cardiac arrest (OHCA) is very poor; only 2–20% of patients can survive till hospital discharge (1–4). The prevalence of pediatric OHCA varies among countries, ranging from 5.4 to 18 per 100,000 person-years (5–7). Patient level characteristics, such as age, sex, and comorbidities might influence OHCA outcome (8–10). For pediatric patients, the prognosis of OHCA is relatively better than that of adult patients, and the rate of survival till hospital discharge ranges from 10 to 20% (6, 7, 11). The relatively poor prognosis of adult OHCA might be due to higher rates of comorbidities, less preserved organ function, and older age. Furthermore, the possible reasons for OHCA in pediatric and adult patients are different. The major reason for adult OHCA is of cardiovascular origin (12), but the major triggers of pediatric OHCA are respiratory problems, sudden infant death syndrome, and trauma (7, 13). The different reasons for OHCA might also impact OHCA outcome.

Pre-hospital factors, such as bystander cardiopulmonary resuscitation (CPR), witnessed OHCA, emergency medical service (EMS) response time, and pre-hospital defibrillation are known prognostic factors of OHCA (14–16). However, for pediatric OHCA patients, pre-hospital airway management remains controversial. Hassen et al. analyzed 1,724 pediatric OHCA cases to compare airway management approaches, and concluded that bag-valve-mask (BVM) ventilation was associated with higher odds of survival till hospital discharge (17); However, Lavonas et al. did not find a statistically significant difference between BVM use and pre-hospital advanced airway management (AAM) (18).

Because the causes of OHCA differ between adults and children, pre-hospital factors might have different effects on the prognosis of adults and children. Although there are studies have focused on pre-hospital factors and survival among pediatric and adult OHCA patients, and the disparities between pediatric and adult patients remain unclear. As a result, the purpose of the study was to (1) evaluate the impact of pre-hospital factors, such as EMS response time, bystander CPR, and pre-hospital defibrillation on outcomes of OHCA, and (2) to analyze the differences in pre-hospital factors in pediatric and adult OHCA.

## Materials and Methods

### Study Design

To evaluate the influence of pre-hospital factors on OHCA, we retrospectively obtained data of OHCA patients from the emergency medical service (EMS) database of Kaohsiung City from January 2015 to December 2019. Kaohsiung is the third largest city in Taiwan with ~2.8 million people (19). The EMS database was reviewed independently by two emergency physicians who were trained and employed at our medical center. The study was approved by our hospital's institutional review board (number: 202100739B0) and was performed in accordance with the ethical standards set forth in the 1964 Declaration of Helsinki and its later amendments. Formal consent from subjects was not required for this type of study.

### Study Setting and Population

The EMS database was described previously (20). Briefly, the EMS is a single-tiered system in Taiwan, with ambulance records stored electronically in every province's EMS command center, which is maintained by the government of Taiwan. In Taiwan, EMS agents can be classified as emergency medical technicians (EMT)-I, EMT-II, and EMT-paramedic (EMT-P) based on the training time that they received and what they are authorized to do (15). Briefly, basic life support (BLS), laryngeal mask airway (LMA), and automated external defibrillator (AED) could be performed by all EMTs, but only EMT-Ps are trained in Advanced Pediatric Life Support and Advanced Cardiovascular Life Support programs conducted in hospitals. Furthermore, some advanced procedures, such as intubation, administration of intravenous medication, and transcutaneous pacing also can be performed by EMT-Ps.

There are two parts in the EMS database of OHCA: (1) that recorded by the EMT and (2) that completed by trained medical record reviewers of hospitals that received the patients. The first part consisted of demographic factors of OHCA patients, such as age, sex, and comorbidities, as well as time, condition, and location of OHCA. Pre-hospital management, such as bystander CPR, airway management, pre-hospital defibrillation using an automated external defibrillator (AED), and EMT-paramedics (EMT-P), are also included in the first part of the EMS database. The second part includes neurological outcomes using the cerebral performance category (CPC) of OHCA patients and patient disposition (21).

Deaths due to trauma, drowning, resuscitation not started because of a pre-ordered “do not resuscitate” (DNR), and missing data or missing records of outcome were excluded after reviewing the EMS database.

### Measurements of Variables and Outcome

Age, sex, and pre-hospital factors, such as EMS response time, pre-hospital defibrillation by AED, reported location of OHCA, initial management by EMTs, and comorbidities, such as malignancy and diabetes, were recorded from the EMS database (22). The primary outcome was patient survival to hospital discharge. The secondary outcome was favorable neurologic outcome, defined as cerebral performance categories (CPC) scale 1–2.

### Statistics

The results of the descriptive analyses of independent variables are reported as the mean ± SD. The age was expressed as medians and first quartile to third quartile (Q1–Q3). Chi-square test, Mann-Whitney U test, and Student *t*-test were used to analyze independent variables. The statistical significance of the relationship between pre-hospital factors, comorbidities, and OHCA outcome was analyzed using binary logistic regression to obtain the odds ratio (OR), 95% confidence interval (CI), and *p*-value for trends. A Kaplan-Meier type plots was used to estimate survival rate following OHCA till four consecutive stages of care (survival for 2 h, survival for 24 h, survival till hospital discharge, and favorable neurologic outcome) (23). The log-rank test was used to calculate the *p*-value for each stage in the Kaplan-Meier type plots. Statistical significance was set at *p* < 0.05. All statistical analyses were performed using IBM SPSS Statistics for Windows, version 25.0 (IBM Corp, Armonk, NY, USA).

## Results

**Figure 1:**
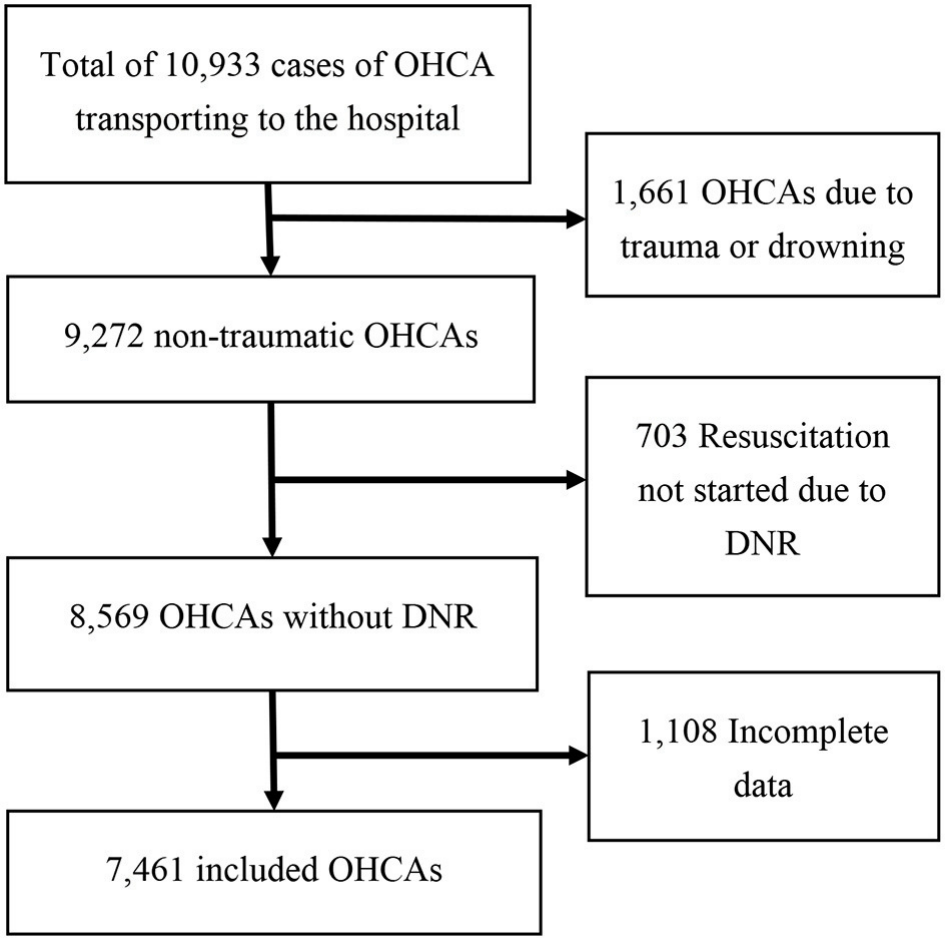
Flow chart of patient disposition in this study. OHCA, out-of-hospital cardiac arrest; DNR, do not resuscitate order.

Figure 1 shows a total of 10,933 cases of OHCA were recorded in Kaohsiung during the study period. Deaths due to trauma or drowning (*n* = 1,661), resuscitation not started due to pre-prescribed DNR order (*n* = 703), and incomplete data (*n* = 1,108) were excluded; finally, 7,461 OHCAs were analyzed in this study.

The demographic characteristics and pre-hospital factors of the pediatric (age ≤ 20 years) and adult patients (age >20 years) are listed in Table 1. There were 124 and 7,337 OHCAs in the pediatric and adult groups, respectively. Pediatric OHCA patients had a higher ratio of public location of cardiac arrest (*p* < 0.001), bystander CPR (*p* = 0.014), and survival till hospital discharge (*p* < 0.001), as well as a lower ratio of pre-hospital defibrillation by AED (*p* = 0.009), laryngeal mask use (*p* < 0.001), diabetes (*p* < 0.001), and malignancy (*p* = 0.011).

**Table 1:** Demographic factors and outcome among pediatric and adult patients of out-of-hospital cardiac arrest patients.

**Characteristics of medical**	**Age ≤ 20**	**Age > 20**	***p***
**out-of-hospital cardiac arrest patients**	***n*** **= 124**	***n*** **= 7,337**	
Age (median, Q1–Q3)	5, 1–17	71, 58–82	<0.001
Male sex	75 (60%)	4,700 (64%)	0.411
EMS response time (min)	7.1 ± 5.6 (6%)	7.1 ± 5.5 (1%)	0.902
Cardiac arrest location (public)	32 (26%)	946 (13%)	<0.001
Attended by EMT-Paramedic	32 (26%)	1,973 (27%)	0.735
Witness	82 (66%)	4,437 (61%)	0.258
Shockable rhythm	2 (2%)	233 (3%)	0.403
Defibrillation by AED	7 (6%)	1,003 (14%)	0.009
Bystander CPR	62 (50%)	2,844 (39%)	0.014
Advanced airway	53 (43%)	6,653 (91%)	<0.001
Diabetes	1 (1%)	1,877 (26%)	<0.001
Malignancy	3 (2%)	702 (10%)	0.01
Survival to hospital discharge	26 (21%)	310 (4%)	<0.001

*EMS, emergency medical service; CPR, cardiopulmonary resuscitation; EMT, emergency medical technicians; AED, automated external defibrillator*.

Table 2 shows the prognostic factors for patients with OHCA. By mean age, patients who survived till hospital discharge were younger (*p* < 0.001). There were higher proportions of male (*p* = 0.02), shorter EMS response time (*p* = 0.004), public location of cardiac arrest (*p* < 0.001), bystander CPR (*p* = 0.004), attended by EMT-P (*p* = 0.003), witness (*p* < 0.001), shockable rhythm (*p* < 0.001), and pre-hospital defibrillation by AED (*p* < 0.001) among patients who survived till hospital discharge. There were significant differences between survival till hospital discharge and mortality in adult patients, but not in pediatric patients in terms of age (*p* < 0.001), sex (*p* = 0.035), EMS response time (*p* = 0.005), bystander CPR (*p* = 0.01), attended by EMT-P (*p* = 0.005), shockable rhythm (*p* < 0.001), and pre-hospital defibrillation by AED (*p* < 0.001).

**Table 2:** Key factors associated with survival till hospital discharge in pediatric and adult out-of-hospital cardiac arrest (OHCA).

	**Overall**			**Age** **≤** **20**			**Age** **>** **20**		
	**Survival to hospital discharge** **(*n* = 336)**	**Mortality** **(*n* = 7,125)**	***p***	**Survival to hospital discharge** **(*n* = 26)**	**Mortality** **(*n* = 98)**	***p***	**Survival to hospital discharge** **(*n* = 310)**	**Mortality** **(*n* = 7,027)**	***p***
Age	59.8 ± 20.6	68.6 ± 17.5	<0.001	8.7 ± 8.0	8.7 ± 8.2	0.993	64.1 ± 14.7	69.4 ± 16.1	<0.001
Male sex	235	4,540	0.02	19	56	0.14	216	4,484	0.035
EMS response time (min)	6.3 ± 2.4	7.2 ± 5.6	0.004	6.3 ± 1.1	7.2 ± 62	0.484	6.3 ± 2.5	7.2 ± 5.6	0.005
Cardiac arrest location (public)	100	878	<0.001	11	21	0.031	89	857	<0.001
Bystander CPR	155	2,751	0.004	14	48	0.659	141	2,703	0.01
Attended by EMT-Paramedic	113	1,892	0.003	9	23	0.248	104	1,869	0.005
Witness	268	4,251	<0.001	21	61	0.076	247	4,190	<0.001
Shockable rhythm	37	198	<0.001	1	1	0.239	36	197	<0.001
Pre-hospital defibrillation by AED	127	883	<0.001	3	4	0.143	124	879	<0.001
Advanced airway	289	6,417	0.016	15	38	0.083	274	6,379	0.156
Diabetes	90	1,788	0.471	1	0	0.052	89	1,788	0.2
Malignancy	29	676	0.61	2	1	0.05	27	675	0.598

*EMS, emergency medical service; CPR, cardiopulmonary resuscitation; EMT, emergency medical technicians; AED, automated external defibrillator*.

Table 3 shows the results of the multivariable logistic regression analysis of survival till hospital discharge of OHCA in different pediatric and adult OHCA groups, adjusted for prognostic confounding factors, including age, sex, EMS response time, public location of cardiac arrest, bystander CPR, attended by EMT-P, witness, shockable rhythm, advanced airway, and pre-hospital defibrillation by AED. For pediatric OHCAs, age (OR = 0.877, 95% CI: 0.764–0.990, *p* = 0.033), public location (OR = 7.681, 95% CI: 1.975–33.428, *p* = 0.003), and advanced airway management (OR = 8.952, 95% CI: 1.414–66.081, *p* = 0.02) were significantly associated with survival till hospital discharge. For adult OHCAs, age (OR = 0.988, CI: 0.980–0.995, *p* = 0.002), EMS response time (OR = 0.907, 95% CI: 0.862–0.951, *p* < 0.001), public location (OR = 1.850, 95% CI: 1.338–2.447, *p* < 0.001), witness (OR = 2.278, 95% CI: 1.699–3.103, *p* < 0.001), shockable rhythm (OR = 1.600, 95% CI:1.042–2.405, *p* = 0.032), and pre-hospital defibrillation by AED (OR = 3.252, 95% CI: 2.473–4.258, *p* < 0.001) were statistically associated with survival till hospital discharge.

**Table 3:** Adjusted odds ratios for survival till hospital discharge in pediatric and adult out-of-hospital cardiac arrest (OHCA).

	**Overall**				**Age ≤ 20**				**Age > 20**			
	**OR**	**95% CI**		***p***	**OR**	**95% CI**		***p***	**OR**	**95% CI**		***p***
Male	0.954	0.74	1.236	0.72	2.276	0.655	9.142	0.2	0.946	0.728	1.237	0.683
Age (one additional year)	0.983	0.98	0.989	<0.001	0.877	0.764	0.99	0.033	0.988	0.98	0.995	0.002
EMS response time (one additional minute)	0.908	0.87	0.951	<0.001	0.978	0.781	1.084	0.766	0.907	0.862	0.951	<0.001
Cardiac arrest location (public)	1.909	1.45	2.494	<0.001	7.681	1.975	33.43	0.003	1.85	1.388	2.447	<0.001
Witness	2.279	1.72	3.074	<0.001	1.495	0.407	6.258	0.55	2.278	1.699	3.103	<0.001
Bystander CPR	1.194	0.95	1.506	0.14	2.019	0.616	7.26	0.249	1.152	0.906	1.464	0.248
Attended by EMT-Paramedic	1.308	1.02	1.667	0.03	2.95	0.845	10.71	0.089	1.283	0.994	1.647	0.056
Shockable rhythm	1.578	1.03	2.363	0.04	2.639	0.052	118.1	0.603	1.6	1.042	2.405	0.032
Pre-hospital defibrillation	3.002	2.29	3.911	<0.001	0.517	0.036	5.047	0.579	3.252	2.473	4.258	<0.001
Advanced airway	0.818	0.58	1.184	0.28	8.952	1.414	66.08	0.02	0.764	0.531	1.13	0.172

*EMS, emergency medical service; CPR, cardiopulmonary resuscitation; EMT, emergency medical technicians; AED, automated external defibrillator*.

Figure 1 shows the crude analysis using the Kaplan-Meier type plots for the pediatric vs. adult group. There was a significant difference between the pediatric and adult groups in survival for 2 h (*p* < 0.001), 24 h (*p* < 0.001), till hospital discharge (*p* < 0.001), and favorable neurologic outcome (*p* < 0.001).

**Figure 2:**
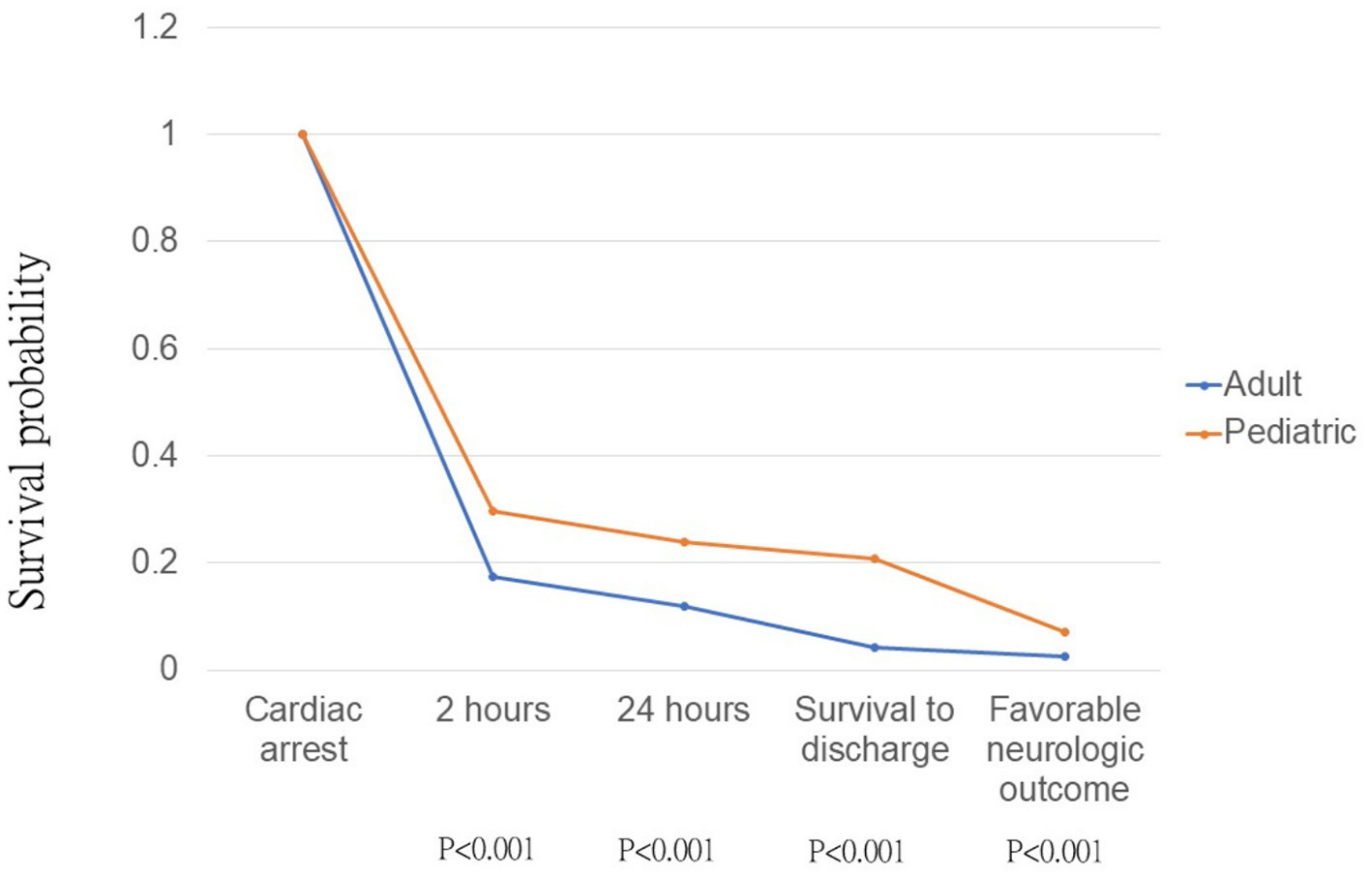
Kaplan-Meier type plots for pediatric and adult out-of-hospital cardiac arrest patients, *p*-value was calculated by log-rank test.

**Figure 3:**
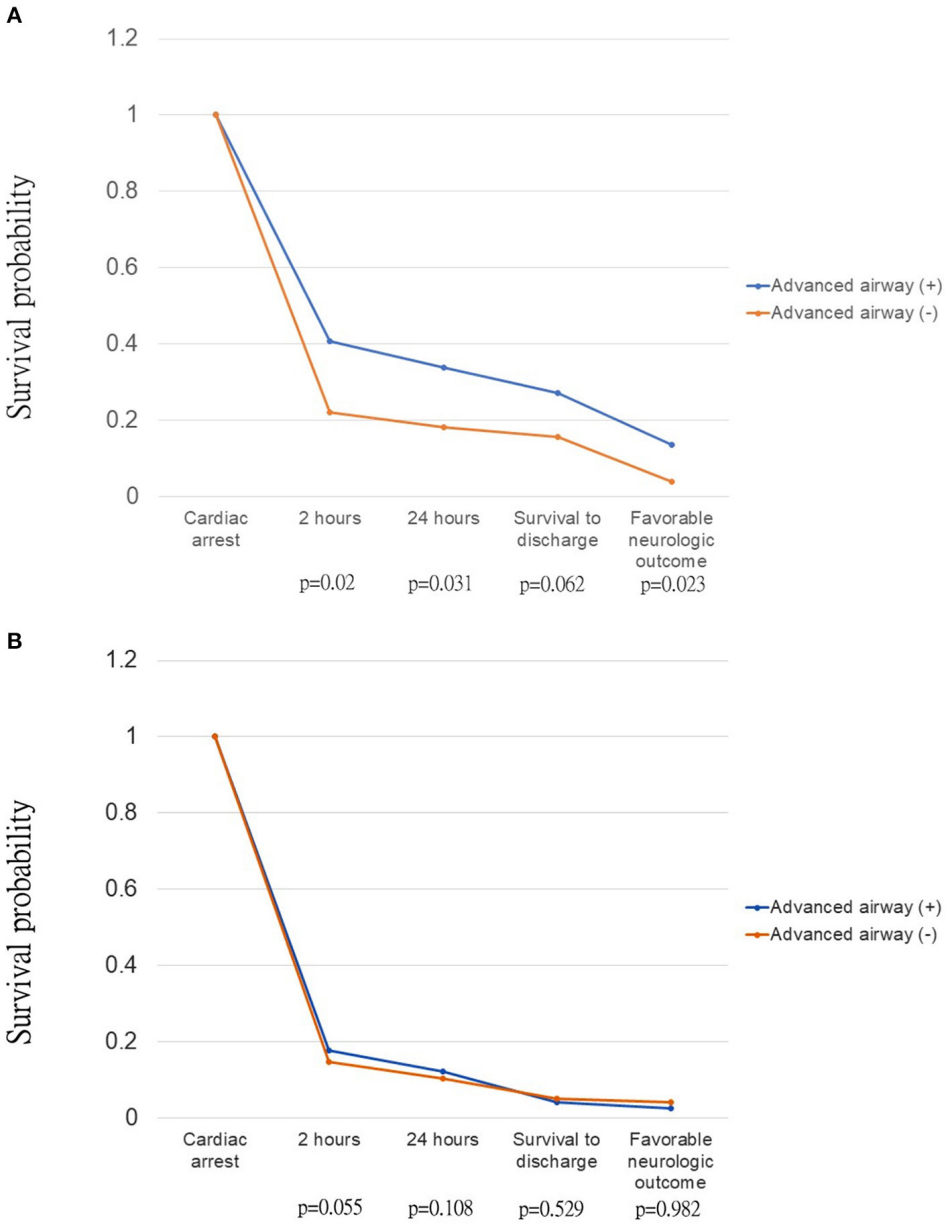
Kaplan-Meier type plots with log-rank test for *p-*value of out-of-hospital cardiac arrest patients, stratified by advanced airway in **(A)** pediatric group; **(B)** adult group.

AAM was an independent prognostic factor for pediatric OHCAs, but not for the adult group. Crude analysis using the Kaplan-Meier type plots for AAM is shown in Figure 2. AAM was associated with improved survival for 2 h (*p* = 0.015), 24 h (*p* = 0.023), and neurologic outcome (*p* = 0.018) in the pediatric group only (Figure 3A), but not in the adult group (Figure 3B).

## Discussion

In this study, we estimated the different prognostic factors for OHCA in adult and pediatric patients. Compared with adult patients, we found prognosis to be better for pediatric OHCA patients, and AAM was an independent prognostic factor in pediatric patients only. Furthermore, age and public location of OHCA were associated with survival in both pediatric and adult patients.

The non-cardiac cause of OHCA, especially asphyxia, is more common in children than in adults (5), thus it is considered that AAM should be a good prognostic factor in pediatric OHCA patients. However, previous studies have shown that pre-hospital AAM has no positive effect on pediatric OHCA survival and neurologic outcomes, including either supraglottic airway or endotracheal intubation (24–26). Ohashi-Fukuda et al. collected data from 2,157 pediatric OHCAs and compared the prognosis for pre-hospital airway management with AAM and BVM, and found no statistically significant difference occurred in OHCA survival rate and neurologic outcome (27). For adult OHCA patients, the influence of AAM in OHCA patients is inconclusive. Benoit et al. showed that the use of endotracheal intubation by pre-hospital EMS was associated with better survival till hospital admission and neurologically when compared to supraglottic airway, but no difference in survival till hospital discharge (28). Compared with pre-hospital AAM, Hasegawa et al. showed that pre-hospital BVM was independently associated with better neurological outcome in adult OHCAs (29). Fouche et al., in a meta-analysis of 17 observational studies, concluded that basic airway intervention is associated with better prognosis than AAM in adult patients (30). The present study showed similar results. In the adult group, pre-hospital AAM was associated with lower odds (OR = 0.764, 95% CI = 0.531–1.130) of survival till hospital discharge, but the difference was not statistically significant. By contrast, in pediatric OHCA, our study found that pre-hospital AAM was independently associated with a higher odd of survival till hospital discharge. This might imply the importance of early AAM in pediatric OHCA due to the relatively common respiratory cause.

In previous studies, regardless of whether they were adults or pediatric patients, OHCA occurring in public areas has a higher probability of a better outcome than that occurring in non-public areas (15, 31). Folke et al. and Hsu et al. showed that adult OHCAs that occurred in residential areas are likely to have longer EMS response times, episodes occurred more often at night, with less shockable rhythm, less witness and bystander CPR, and later time for initiating CPR and first defibrillation (15, 31). Shida et al. also showed better outcomes in pediatric OHCA occurring in public areas, which is related to a higher probability of witness bystander CPR, shocked by public AEDs, and shorter EMS response time (32). The present study also supports that OHCA occurring in a public location is associated with better prognosis, both in pediatric and adult patients.

Previous studies have revealed that bystander CPR is a prognostic factor for OHCA, both in adults and children (10, 16, 33, 34). Luc et al. collected data from the French OHCA registry and demonstrated that bystander CPR is associated with a better 30-day survival rate (35). In the pediatric population, Law et al. also found that bystander CPR is associated with survival till hospital discharge (6). Kiyohara et al. also revealed that bystander CPR is associated with favorable neurologic outcomes (33). However, the influence of bystander CPR on survival till hospital discharge was not statistically significant in the current study. One possible reason is that the populations included were different. Traumatic OHCAs were not excluded in Law et al.'s (6) and Luc et al.'s (35) studies, whereas Kiyohara et al. (33) only included pediatric patients aged 6–17 years. In our study, bystander CPR accounts for 50% in pediatric group and 39% in adult group, but it didn't demonstrate the better OHCA survival rate in both groups. This might be explained by the poor quality of bystander CPR or delayed bystander CPR. The time window between collapse to CPR initialized and the quality of bystander CPR were not well-documented in our database. However, no-flow interval (time from collapse to the initiation of CPR) and CPR quality might impact the outcome of OHCA (36).

Early defibrillation for shockable rhythm is crucial for pediatric OHCA patients and is related to better outcomes. In a study by Kiyohara et al. (33), pediatric OHCA was related to better outcomes (survival rate and favorable neurologic outcome) when OHCA occurred with bystander CPR and the use of public AED. Similar results were observed by Fukuda et al. (24); however, they also reported that using public AEDs is not related to better OHCA neurologic outcomes in the unwitnessed or non-cardiac etiology subgroup. In our study, the results showed no significant difference between shockable rhythm and survival till hospital discharge. This might be explained by the lower number of cases included in our study (only seven pediatric cases that received pre-hospital defibrillation). Another possible explanation is that most pediatric OHCA cases are non-cardiogenic; thus, there was no statistically significant difference between AED use and better OHCA outcomes (5, 24). Further studies are required to clarify this. However, increased public AED is still important for pediatric OHCA due to sports-related OHCA being more common in school-age children, and is more likely to be related to witness, bystander CPR, and idiopathic ventricular fibrillation (37).

As for EMS response time, many previous studies have shown good OHCA outcomes with shorter EMS response time. In pediatric OHCA studies, Nehme et al. showed that a shorter EMS response time was not related to OHCA event survival rate (38). However, if OHCA cases survive, a shorter EMS response time was related to the survival till hospital discharge rate. Chang et al. showed better neurologic outcomes with shorter EMS response time (39); but even with delayed EMS response time, this result was still preserved when there was bystander CPR with dispatcher assistance. In our study, the EMS response time was related to higher odds of survival till hospital discharge for adults, but the difference was not statistically significant for pediatric patients. This might be explained by the fact that pediatric OHCA outcomes are related to EMS response with appropriate bystander CPR and dispatcher assistance CPR. However, our results showed no significant relationship between bystander CPR and better survival till discharge rate, and the result of EMS response may also be affected. Further studies are required to clarify this issue in the future.

There are some limitations to the present study. First, this study was conducted in a retrospective manner; therefore, we could not identify any causality. Second, because our cases included those that were sent to the hospital by the EMS, some OHCA patients were not included if they were sent by their family, cohabiting partners, or residential facilities. Third, the database was limited to Kaohsiung area only; therefore, the results of our study might not be applicable to other areas due to different medical level facilities or cultural gaps. Fourth, our data did not include extracorporeal CPR and dispatcher-assisted CPR. Finally, post-resuscitation care, such as target temperature management and level of hospital receiving OHCA patients, were not included in the database.

## Conclusion

There were variations between pediatric and adult patients with OHCA in prognostic factors. The prognosis of the pediatric group was better than that of the adult group. Furthermore, advanced airway use was an independent prognostic factor in pediatric patients, but not in adult patients. Age and public location of OHCA were independently associated with survival till hospital discharge in both pediatric and adult patients in Kaohsiung City in Taiwan.

## Data Availability Statement

The original contributions presented in the study are included in the article/supplementary material, further inquiries can be directed to the corresponding author/s.

## Ethics Statement

The studies involving human participants were reviewed and approved by Kaohsiung Chang Gung Memorial Hospital Institutional Review Board (number: 202100739B0). Written informed consent from the participants' legal guardian/next of kin was not required to participate in this study in accordance with the national legislation and the institutional requirements. Written informed consent was obtained from the individual(s), and minor(s)' legal guardian/next of kin, for the publication of any potentially identifiable images or data included in this article.

## Author Contributions

F-JC designed this study, coordinated data collection, data analysis, and carefully revised the manuscript. W-TW contributed to drafting the manuscript and submitted the final version. S-CH and Y-NH contributed to drafting the manuscript. M-TT contributed to the design of the study and supported data collection and analysis. I-MC conducted the quantitative data analysis and approved the final version. K-HW provided insight into the design of the study and critically revised the manuscript. All authors contributed to the article and approved the submitted version.

## Conflict of Interest

The authors declare that the research was conducted in the absence of any commercial or financial relationships that could be construed as a potential conflict of interest.

## Publisher's Note

All claims expressed in this article are solely those of the authors and do not necessarily represent those of their affiliated organizations, or those of the publisher, the editors and the reviewers. Any product that may be evaluated in this article, or claim that may be made by its manufacturer, is not guaranteed or endorsed by the publisher.
